# Short- and long-term outcomes of rectal cancer patients with high or improved low ligation of the inferior mesenteric artery

**DOI:** 10.1038/s41598-020-72303-0

**Published:** 2020-09-18

**Authors:** Chenghai Zhang, Lei Chen, Ming Cui, Jiadi Xing, Hong Yang, Zhendan Yao, Nan Zhang, Fei Tan, Maoxing Liu, Kai Xu, Xiangqian Su

**Affiliations:** Key Laboratory of Carcinogenesis and Translational Research (Ministry of Education), Department of Gastrointestinal Surgery IV, Peking University Cancer Hospital & Institute, 52 Fu-Cheng Road, Hai-Dian District, Beijing, 100142 China

**Keywords:** Cancer therapy, Gastrointestinal cancer

## Abstract

The ligation site of the inferior mesenteric artery (IMA) during laparoscopic radical resection for rectal cancer has been controversial. Consecutive patients (n = 205) with rectal cancer who underwent laparoscopic-assisted low anterior resection from January 2009 to December 2015 were retrospectively analyzed. The patients were divided into high ligation (n = 126) and improved low ligation groups (n = 79). A total of 205 rectal cancer patients underwent laparoscopic assisted anterior resection: 126 patients in the high ligation group and 79 patients in the improved low ligation group. The improved low ligation group was better than the high ligation group in terms of postoperative flatus time and postoperative defecation time. There were no differences between the groups in terms of blood loss, operation time, total number of lymph nodes, anastomotic leakage, postoperative time to first liquid diet and postoperative hospital stay. There were also no differences in 5-year overall survival (OS). Compared to high ligation, the improved low ligation ensures the extent of lymph node dissection, and promotes the early recovery of postoperative gastrointestinal function, but does not increase the operation time, bleeding risk, or anastomotic leakage. A ligation site of the IMA in laparoscopic rectal cancer surgery may not influence oncological outcomes.

## Introduction

The efficacy and safety of laparoscopic radical surgery for rectal cancer have been confirmed^1,2^. The principle of total mesenteric resection for rectal carcinoma and the protection of pelvic autonomic nerves have achieved consensus worldwide. However, the ligation site of the IMA in laparoscopic radical resection for rectal cancer is still being debated^3–5^.

In laparoscopic rectal cancer surgery, the ligation site of the IMA can be divided into a high ligation site , where the IMA is ligated at its origin from the aorta and the traditional low ligation site, which is below the branch of the left colon artery (LCA). In fact, these two ligation methods have their own advantages and disadvantages. A high ligation site helps to improve surgical radicalization and achieve more accurate staging, and can reduce anastomotic tension^6–8^. However, it may lead to poor blood flow in the anastomosis, resulting in an increased risk of anastomotic leakage^9^. Traditional low ligation may reduce anastomotic leakage, due to increased blood flow to the proximal end of the anastomosis^3,10^. However, this type of ligation may affect long-term survival because of the absence of upward lymph node dissection.

Japanese guidelines recommend that the lymph nodes around the root of the IMA should be dissected for clinical T2 or higher stage rectal cancer. However, traditional low ligation does not meet this requirement. Therefore, some surgeons have improved the traditional low ligation procedure and have performed lymph node dissection up to the root of the IMA with preservation of the LCA (improved low ligation)^11–13^. Therefore, the main purpose of the present study is to compare the short-term and long-term results of high ligation and improved low ligation.

## Results

### Patient characteristics

The clinical data of 205 rectal cancer patients who underwent potentially curative laparoscopic resection are shown in Table 1. There were no serious surgical complications (such as intraoperative major bleeding and ureteral injury) or perioperative deaths in either group. There were no statistically significant differences in tumor location, age, sex, body mass index, tumor size, T stage, TNM stage, preoperative CRT, or prophylactic ileostomy between the two groups. Preoperative chemotherapy was performed in 19 patients and 10 patients in the high ligation group and improved low ligation group, respectively.

**Table 1:** Clinical data of the 205 rectal cancer patients who underwent laparoscopic-assisted low anterior resection.

Category		High ligation (n = 126)	Low ligation with LND (n = 79)	*p* Value
Age (years)*		60.3 ± 10.7	61.3 ± 10.2	0.474
**Sex**				0.163
Male		64(50.8%)	48(60.8%)	
Female		62(49.2%)	31(39.2%)	
**Body mass index (kg/m**^**2**^**)**				0.532
≥ 25		44(34.9%)	31(39.2%)	
< 25		82(65.1%)	48(60.8%)	
Tumor size (cm)*		4.3 ± 1.8	3.9 ± 1.7	0.096
**Tumor location**				0.57
Lower		31(24.6%)	10(12.7%)	
Middle		71(56.3%)	47(59.5%)	
Upper		24(19.1%)	22(27.8%)	
**T stage**				0.412
T1		7(5.6%)	8(10.1%)	
T2		23(18.3%)	14(17.7%)	
T3		75(59.5%)	46(58.2%)	
T4		21(16.6%)	11(14.0%)	
**TNM stage**				0.633
I		22(17.5%)	17(21.5%)	
II		40(31.7%)	27(34.2%)	0.202
III		64(50.8%)	35(44.3%)	
**Preoperative CRT**				0.707
Yes		19(15.1%)	10(12.7%)	
No		107(84.9%)	69(87.3%)	
**Prophylactic ileostomy**				0.626
Yes		64(50.8%)	38(48.1%)	
No		62(49.2%)	41(51.9%)	

### Clinical data related to surgery

The median operation time was 173 min in the high ligation group and 180 min in the modified low ligation group (*p* = 0.680). There was no significant difference in the operation time between the two groups. Additionally, the median intraoperative blood loss in the high ligation group was 30 ml (30.0–50.0 ml), which was not significantly different from that in the modified low ligation group 50.0 ml (30.0–100.0 ml). The mean number of retrieved lymph nodes per patient in the high ligation group was 16.4 and was 14.7 in the modified low ligation group, with no statistically significant differences between the two groups. There was also no significant difference in anastomotic leakage between the two groups, but only 2 cases occurred in the high ligation group (Table 2).

**Table 2:** Effect of two kinds of surgical methods on the operation quality.

Surgical data	High ligation (n = 126)	Low ligation with LND (n = 79)	*p* Value
Operation time, min^a^	173.0(90.0–368.0)	180.0(93.0–435.0)	0.680
Blood loss, ml^a^	30.0(10.0–400.0)	50.0(10.0–600.0)	0.121
Number of harvested LN^b^	16.4 ± 7.5	14.7 ± 6.8	0.112
Anastomotic leakage	2	0	0.524

*LND* lymph node dissection around the root of the inferior mesenteric artery.

^a^Median (range).

^b^Mean ± standard deviation.

### Postoperative recovery

First exhaust and defecation are generally used as indicators of postoperative gastrointestinal function recovery. The modified low ligation group was significantly earlier than the high ligation group in terms of first exhaust time (*p* = 0.007) and first defecation time (*p* = 0.016). There was no significant difference between the two groups in the postoperative hospital stay (*p* = 0.564) (Table 3).

**Table 3:** Effect of two kinds of operations on early recovery of gastrointestinal function and postoperative hospital stay.

Postoperative features	High ligation (n = 126)	Low ligation with LND (n = 79)	*p* Value
Time to first flatus, days^a^	4.5 ± 2.0	3.8 ± 1.9	0.007
Time to first defecation, days^a^	5.3 ± 2.2	4.5 ± 2.1	0.016
Postoperative hospital stay, days^a^	9.5 ± 4.5	9.1 ± 3.9	0.564

*LND* lymph node dissection around the root of the inferior mesenteric artery.

^a^Mean ± standard deviation.

### Long-term results

**Figure 1:**
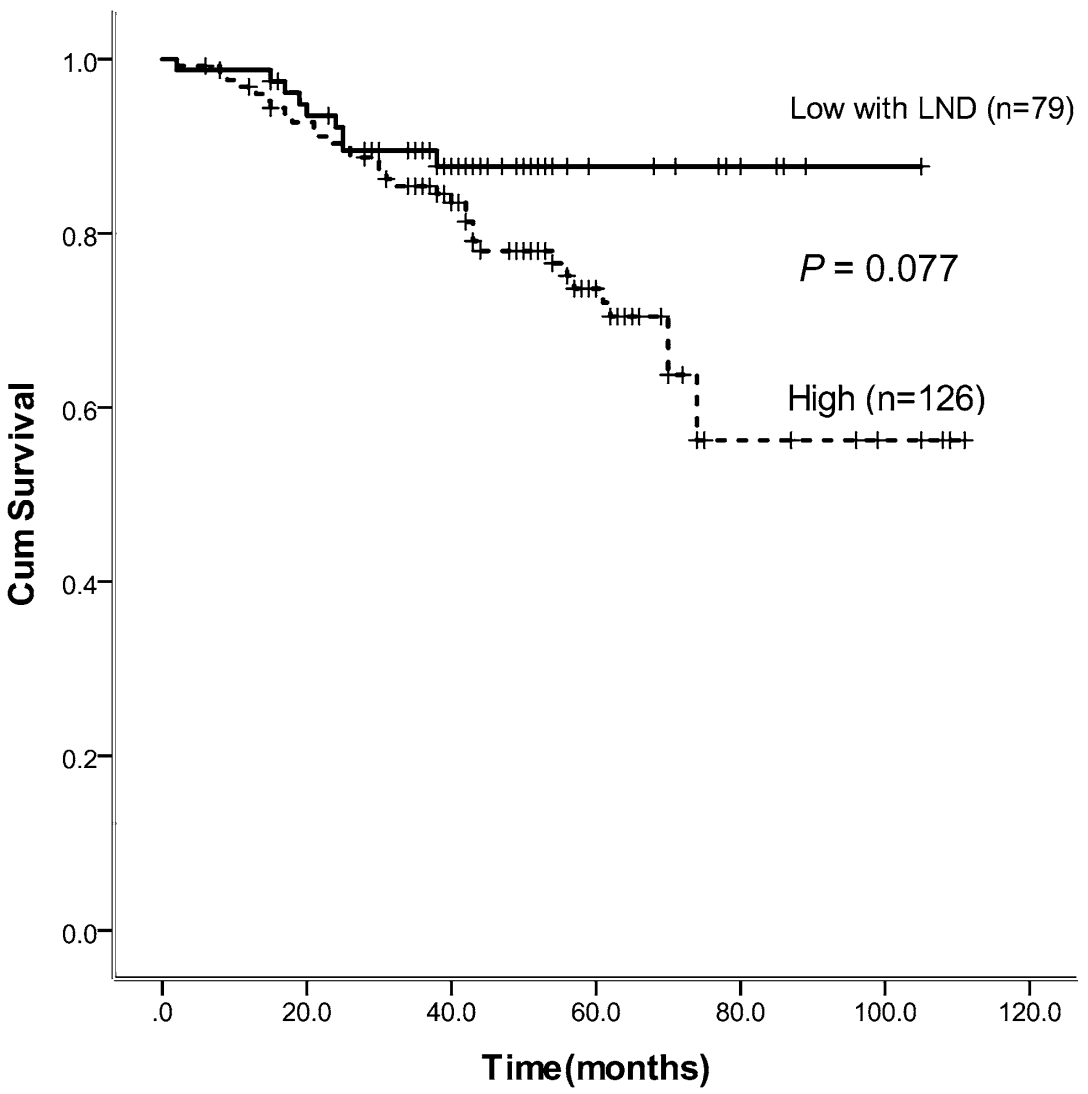
There was no significant difference in 5-year overall survival between the groups (*p* = 0.077).

The median time from operation to follow-up was 52 months (range 1–121 months). The 5-year overall survival rate did not differ significantly between the high- and improved low -ligation groups (78.1 versus 87.7%, respectively; *P* = 0.077). Survival curves, using the Kaplan -Meier method, are presented in Fig. 1.

## Discussion

Advanced rectal cancer or sigmoid colon cancer has a higher rate of lymph node metastasis at the root of the inferior mesenteric artery. The Japanese Society for Cancer of the Colon and Rectum (JSCCR) suggested that D3 dissection should be performed for advanced rectal cancer, because 1.4% of pT2 rectal cancer cases were accompanied by No.253 lymph node metastases^14^. However, it is difficult to dissect No.253 lymph nodes completely with preservation of the LCA under laparoscopic surgery due to the complexity of the operation. Typical lymph nodes are relatively easy to remove with high ligation, but there are concerns about the blood supply of the anastomotic stoma. Therefore, improved low ligation (preservation of the LCA and complete dissection of No.253 lymph nodes) is an ideal surgical method, but its safety and effectiveness need to be further validated.

In the present study the ligation site of the IMA in rectal cancer surgery did not affect the anastomotic leakage rate, similar to previous research^15,16^. There were only two cases of anastomotic leakage in the high ligation group, but there were no cases in the improved low ligation group. One of the two patients with anastomotic leakage was a 70-year-old male who underwent preoperative radiotherapy and had diabetes. The other was a 65-year-old man with diabetes, hypertension and atherosclerotic disease. The lower incidence of anastomotic leakage is partly due to the higher ileostomy rate. Several studies^17,18^ have shown that a temporary ileostomy following low anterior resection for rectal cancers can reduce AL and promote the recovery of AL. As is well known, the main cause of anastomotic leakage is anastomotic tension and reduced blood supply. For the two patients, although high ligation could relieve the anastomotic tension, it may have reduced blood flow near the anastomosis. Several studies^10,19^ have shown that colic blood flow in the vicinity of the anastomosis is decreased in high ligation groups compared with low ligation groups, even with ischemia or necrosis of the proximal bowel^20,21^. In addition, the use of high ligation of the IMA might cause mesenteric ischemia. A recent study from Sweden^22^ reported that the incidence of mesenteric ischemia was associated with high arterial ligation during rectal cancer surgery. By contrast, low ligation ensures adequate blood supply to the proximal limb of the anastomosis due to the retention of the LCA. Several reviews^9,23^ have also shown that low ligation is associated with a lower rate of anastomotic leakage.

The slightly longer operation time in the improved low -ligation group may reflect the increased operational complexity. It is a very difficult step to completely dissect apical lymph nodes around the root of the IMA with preservation of the LCA in laparoscopic rectal cancer surgery, especially for obese patients. Compared to high ligation, improved low ligation requires more operation steps and more skilled cooperation. Furthermore, the variation in the origin of the LCA from the IMA also increases the complexity of surgery^24,25^. However, with the improvement of surgical techniques, the operation time of this step could be significantly shortened, so the difference between the two groups was not statistically significant. In addition, there was no significant difference in the amount of blood loss between the two groups, because most of the operative procedures were the same except for retention of the LCA. For skilled surgeons, dissection of No. 253 lymph nodes with preservation of the LCA does not significantly increase the risk of surgery and additional bleeding.

The postoperative time to first flatus and defecation was slightly shorter in the improved low ligation group than in the high ligation group (*p* < 0.05). Adequate blood supply and perfect autonomic function are important factors in the recovery of postoperative gastrointestinal function. High ligation of the IMA leads to a decrease in anastomotic blood perfusion^19,26^, which may affect anastomotic healing and functional recovery. The left paraaortic trunk runs along the left side of the aorta, passes over the posterior wall of the IMA and joins with the right trunk to compose the superior hypogastric plexus^27^. Therefore, it is not easy to damage the nerve trunk during ligation of the IMA below the level of the LCA. Ferguson et al. ^28^also found that high ligation may increase the risk of autonomic dysfunction around the root of the IMA. Admittedly, the mean time of postoperative first flatus and defecation was less than one day in the improved low ligation group compared with the high ligation group. Although there was a statistically significant difference, its clinical value was limited.

No significant difference was found in the total number of lymph nodes removed or the long-term survival rate (5-year OS and 5-year DFS) between the two groups, in agreement with previous studies^3,9,15,16,23,29,30^. The modified low ligation method is different from the traditional method and does not reduce the range of lymph node dissection. Conventional low ligation is defined as ligation of the IMA below the level of the LCA and removal of lymph nodes that lie along the IMA trunk (from the origin of the LCA to the bifurcation of the superior rectal artery) without dissection of No. 253 nodes^29^. The modified low ligation technique not only preserved the LCA but also completely removed the lymph nodes at or around the root of the IMA. Therefore, from an anatomical perspective, the range of lymph node dissection of improved low ligation is equivalent to that of high ligation.

Although the results of this study have certain clinical value, there are still several limitations. First, the retrospective nature of the study resulted in some statistical bias. The site of IMA ligation was determined by the surgeons during the operation rather than assigned randomly before surgery, which might have influenced the reliability of the results. Therefore, a randomized controlled trial (RCT) on this issue should be performed in the future. Second, the sample size was small. Cardiovascular disease is a confounder of the study results. However, patients with cardiovascular disease were not discussed separately mainly due to the small sample size. Third, the improved low ligation technique was more helpful than high ligation for the recovery of postoperative gastrointestinal function. The reliability of this conclusion needs further verification by RCTs with large sample sizes.

## Conclusion

Compared to high ligation, improved low ligation ensures the extent of lymph node dissection, and promotes the early recovery of postoperative gastrointestinal function, but does not increase the operation time, bleeding risk, or anastomotic leakage. A ligation site of the IMA in laparoscopic rectal cancer surgery may not influence oncological outcomes.

## Patients and methods

### Patients

The study subjects were 205 patients who consecutively underwent laparoscopic-assisted low anterior resection (LAR) for rectal cancer between January 2009 and December 2015. According to the IMA ligation site, these patients were divided into a high ligation group (126 cases) and an improved low ligation group (79 cases). The study inclusion criteria were as follows: patients with pathologically confirmed adenocarcinoma, tumors located less than 15 cm from the anal verge, patients with cTNM stage I-III, and patients who had undergone laparoscopic-assisted LAR. Patients with other malignant tumors, distant metastases, previous major abdominal surgery, palliative surgery or transanal local resection were all excluded. All treatment methods performed in this study were in accordance with the colorectal cancer guidelines of the Chinese Society of Clinical Oncology (CSCO) and the National Comprehensive Cancer Network (NCCN). The experimental protocols were approved by the ethical standards of the Ethics Committee of Peking University Cancer Hospital & Institute and in accordance with the 1964 Helsinki declaration. Informed consent was obtained from each patient enrolled in the study.

### Types of IMA ligation

**Figure 2:**
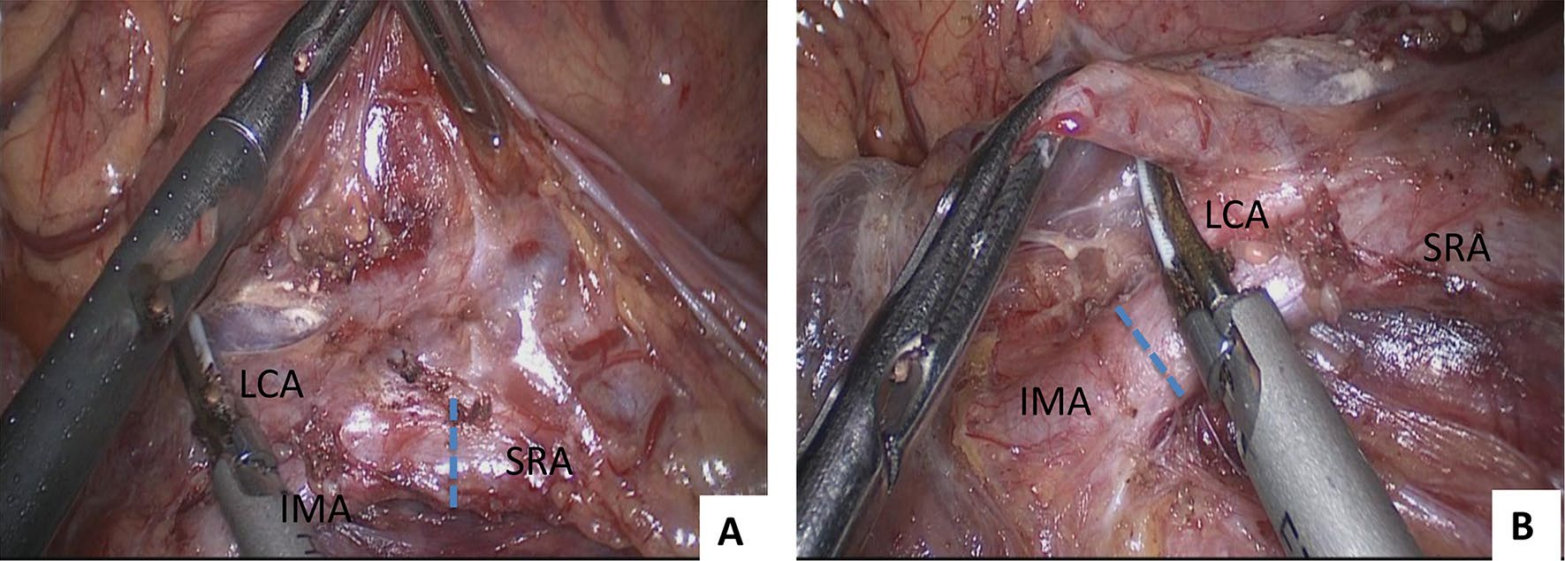
Surgical image of improved low ligation and high ligation of the inferior mesenteric artery. (**A**) Improved low ligation, the dashed line indicates the ligation site of the SRA; (**B**) high ligation, the dashed line indicates the ligation site of the root of the IMA. *IMA* inferior mesenteric artery; *IMV* inferior mesenteric vein; *LCA* left colic artery; *SRA* superior rectal artery.

In the improved low ligation group, the lymph nodes with adipose tissue from the root of the IMA to the bifurcation of the superior rectal artery (SRA) and the LCA were carefully dissected and the artery wall was clearly exposed. Then, the LCA was dissected from its origin to the left until the inferior mesenteric vein (IMV) was recognized. The extraction of lymph nodes was continued along the IMV up to the same level of the origin of the IMA. Subsequently, the IMV was ligated at the level of the root of the IMA, and the IMA was ligated below the LCA, with preservation of the LCA. Therefore, the adipose tissues with lymph nodes in the area surrounded by the IMV, IMA and LCA were removed entirely (Fig. 2A).

In the high ligation group, the lymph nodes and adipose tissues around the root of the IMA were completely dissected. Subsequently, the root of the IMA was cut 1 cm from the abdominal aorta. Then, the IMV was found and ligated at the same level (Fig. 2B).

The remaining surgical procedures, such as laparoscopic total mesorectal excision, autonomic nerve protection and colorectal/colo-anal anastomosis were completely consistent.

### Observation indexes

Indexes associated with operation quality: operation time, intraoperative blood loss, number of harvested lymph nodes and anastomotic leakage.

Indexes associated with early recovery of postoperative gastrointestinal function: time to first flatus, time to first defecation, and postoperative hospital stay.

### Definition of rectal anastomotic leakage

Anastomotic leakage (AL) was defined as a defect of the intestinal wall at the anastomotic site resulting in communication between the intra- and extraluminal compartments^31,32^. Patients with AL often had accompanying fever, abdominal pain, and other symptoms of pelvic infection. AL was usually confirmed by pelvic CT, colonoscopy or intraoperative exploration.

### Follow-up

All postoperative complications were recorded in detail. Physical examinations, serum carcinoembryonic antigen (CEA) tests and serum carbohydrate antigen 19–9 (CA19-9) tests were conducted every 3 months for 2 years, and then every 6 months for 2–5 years. Chest, abdominal and pelvic computed tomography was carried out every 6–12 months for a total of 5 years. Colonoscopy was performed one year after surgery and, repeated at 3 years, and then every 5 years. All patients underwent postoperative follow-up for at least 5 years.

### Statistical analysis

Continuous variables are described as the mean (s.d.) values. Categorical variables are presented as frequencies and percentages. Student^'^s *t* test and χ^2^ test were used to analyze continuous and categorical variables respectively. Survival curves were generated by the Kaplan–Meier method, and the significant difference between the high- and low ligation groups was established with the log-rank test. A *P*-value < 0.05 was considered statistically significant. All analyses were carried out with SPSS version 22.0 (SPSS, Inc., Chicago, IL, USA).

### Ethical approval

All treatment methods performed in this study were in accordance with the colorectal cancer guidelines of the Chinese Society of Clinical Oncology (CSCO) and National Comprehensive Cancer Network (NCCN). The experimental protocols were approved by the ethical standards of the Ethics Committee of Peking University Cancer Hospital & Institute and in accordance with the 1964 Helsinki declaration. Informed consent was obtained from each patient enrolled in the study.

## Data Availability

The data used and analyzed in the present study are available from the corresponding author on reasonable request.
